# Metabolomics of Aurantio-Obtusin-Induced Hepatotoxicity in Rats for Discovery of Potential Biomarkers

**DOI:** 10.3390/molecules24193452

**Published:** 2019-09-23

**Authors:** Longlong Xu, Jian Li, Xianglin Tang, Yuguang Wang, Zengchun Ma, Yue Gao

**Affiliations:** 1 College of Life Science and Bioengineering, Beijing University of Technology, Beijing 100124, China; xulonglong1020@163.com; 2 Department of Pharmaceutical Sciences, Beijing Institute of Radiation Medicine, Beijing 100850, China; lijianjky@163.com (J.L.); tangxianglin@139.com (X.T.); wyg79@163.com (Y.W.); mazchun@139.com (Z.M.)

**Keywords:** aurantio-obtusin, anthraquinone, liver injury, metabolomics

## Abstract

Aurantio-obtusin is an anthraquinone derived from *Cassia obtusifolia* (cassiae semen). It is also used as a tool and a detection index for the identification of cassiae semen, as stipulated by the Chinese Pharmacopoeia. Anthraquinones, the main components in cassiae semen, have been reported to show hepatotoxicity. This study investigates the hepatotoxicity of aurantio-obtusin in male Sprague–Dawley rats. We randomly divided the animals into a blank control group and treated three test groups with different doses of aurantio-obtusin: Low dose (4 mg/kg), medium dose (40 mg/kg), and high dose (200 mg/kg). Each group was treated with aurantio-obtusin for 28 days, whereas the control group was administered an equal volume of 0.5% carboxymethyl cellulose sodium salt (CMC-Na) aqueous solution. Subsequently, we conducted biochemical, hematological, and pathological investigations and determined the weight of different organs. We used serum metabolomics to identify possible biomarkers related to hepatotoxicity. The low-dose group showed no significant liver injury, whereas the medium- and high-dose groups manifested obvious liver injury. Compared with the control group, the test groups showed an increase in alanine transaminase, aspartate transaminase, and alkaline phosphatase levels. The liver organ coefficient also significantly increased. Additionally, we found significant changes in the hematological indices. Metabolomics analysis showed that aurantio-obtusin induced 28 endogenous markers related to liver injury. Our data indicate that aurantio-obtusin induces hepatotoxicity in rat liver in a dose-dependent manner and is mediated by pathways involving bile acids, fatty acids, amino acids, and energy metabolism. In particular, changes in bile acid content during treatment with therapeutic agents containing aurantio-obtusin deserve increased attention.

## 1. Introduction

Traditional Chinese medicines (TCMs) are a national treasure, with a 5000-year history, and are characterized by remarkable clinical efficacy and few adverse reactions. However, it is a challenge to develop TCMs for worldwide acceptance [1,2]. The material basis of TCMs is unknown because of the lack of specific diagnostic indicators. Therefore, the combined use of Chinese and Western medicine is complex. As a result, the safety of TCMs cannot be guaranteed, which in turn limits its clinical applications. For example, the Berberine Incident [3,4] in Singapore and the incident of *Polygonum multiflorum* [5,6,7,8] in China are well known, and these incidents have adversely affected the safety and clinical application of TCMs. Despite historical insights into the safety and nontoxicity of TCMs, medicinal herbs intended for clinical and food applications should be investigated in depth. Generally, these medicinal herbs are mild and exhibit few adverse reactions, suggesting their possible use in clinical settings or in daily life. However, safety issues are often overlooked and are more likely to involve potential toxicity. Preclinical studies are used for the safety evaluation of TCMs [9,10].

Cassiae semen is one of the plant species with extensive medicinal and food applications that has been documented by the National Health Commission of the People’s Republic of China. Clinical studies have shown that treatment with cassiae semen clears the liver, improves vision, alleviates catharsis, reduces blood pressure and blood lipids, boosts immunity, and promotes bacteriostasis [11,12,13,14]. As a dietary ingredient, cassiae semen is also used as a supplement in porridges and health beverages, suggesting its role as a food and medicine with abundant active ingredients. Cassiae semen is rich in proteins, polysaccharides, vitamins, and minerals and contains chrysophanol, emodin, aloe emodin, rhein, emodin methyl ether, aurantio-obtusin, and other anthraquinones. Anthraquinones are the main active ingredients in many TCMs, such as rhubarb, *Polygonum multiflorum*, aloe, and senna leaf [15,16]. However, these anthraquinone compounds have been associated with safety issues. Cases of adverse reactions in clinics have been reported. The use of cassiae semen in combination with the herbal medicines previously mentioned increases anthraquinone levels, which may promote or aggravate adverse reactions. Whether anthraquinone-containing TCM is safe for long-term human consumption has not yet to be established, which has become a basic issue in TCMs and the health food industry. Studies have shown that anthraquinones in cassiae semen can trigger subchronic toxicity in the human body and has a significant negative impact on the liver, kidney, and digestive and reproductive systems, and even induces pathological changes [17]. These side effects also hinder its processing and utilization. Currently, studies on the toxicity of cassiae semen are limited. Therefore, the safety of specific anthraquinone components warrants further investigation to comprehensively resolve its safety issues.

Aurantio-obtusin is a free, unique anthraquinone compound, isolated from the effective fraction of cassiae semen that has anti-inflammatory [18,19] and lipid-lowering activities [20]. Since the publication of the 2005 edition of the Chinese Pharmacopoeia, aurantio-obtusin has been used as a quality control standard instead of rhein. Pharmacokinetic analysis showed a rapid distribution of aurantio-obtusin in animals, whereas elimination is slow [21]. According to second-order kinetics, aurantio-obtusin exhibits a shorter peak time and better absorption, suggesting that aurantio-obtusin is a safe and effective lipid-lowering drug warranting further validation studies [22].

Chrysophanol is a common active ingredient of rhubarb, *P. multiflorum, Polygonum cuspidatum*, and aloe. Ethanol concentration has a significant effect on the extraction, separation, and purification of aurantio-obtusin from cassiae semen. The compound is slightly soluble in water; however, cassiae semen water extract is generally used in clinics. Ultimately, we selected the specific anthraquinone of cassiae semen to investigate the toxicity of aurantio-obtusin in animals based on metabolomics and effectively analyzed various metabolic pathways. Using metabolomics, we detected the dynamic changes and trends of serum maps to analyze the metabolic pathways and searched for biomarkers related to hepatotoxicity of aurantio-obtusin. We analyzed its regulatory effects on endogenous metabolites to determine the biomarkers to facilitate early clinical screening for potential toxicity.

## 2. Results

### 2.1. Body Weights and Organ Coefficient

**Figure 1:**
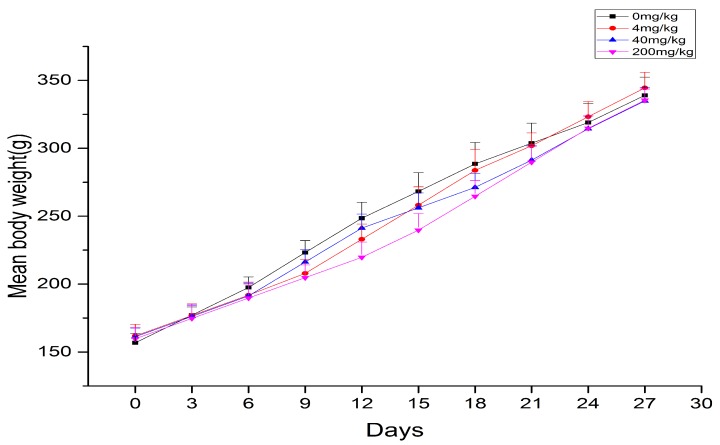
The mean body weight of rats in each group exposed to continuous treatment with aurantio-obtusin (x ± s, *n* = 8).

During the duration of aurantio-obtusin treatment, the physiological characteristics of each rat remained the same as those of the control group, and intake and water consumption were normal. No obvious symptoms of poisoning or death occurred. In this study, the body weight of rats increased with prolonged administration. Although the average body weight of rats in the blank control group was slightly higher than that in the aurantio-obtusin-treated group, the weight of all of the rats did not significantly change (Figure 1).

After administration, the liver tissues of rats in the high-dose group differed from those of the control group, suggesting possible pathological changes. Compared with the control group, the absolute and relative weights of the liver in the administration group increased (*p* < 0.05, *p* < 0.001); however, no significant difference was detected in the low-dose group (Table 1). The results showed that the medium and high doses of aurantio-obtusin had significant effects on the liver organ coefficient, which was calculated as follows: Organ coefficient = liver weight/body weight × 100%.

### 2.2. Toxicological Examination

The hematological data are shown in Table 2. After 28 days of continuous treatment with aurantio-obtusin, we observed a slight decrease in hemoglobin (HGB) levels and hematocrit (HCT) in rats belonging to the 40 and 200 mg/kg groups (*p* < 0.05, *p* < 0.01) and observed a decrease in the number of white blood cells (WBCs) in rats treated with 4 and 40 mg/kg aurantio-obtusin (*p* < 0.05, *p* < 0.01). The number of neutrophils (%) significantly increased in the 4 mg/kg aurantio-obtusin group (*p* < 0.05), which was accompanied by a significant decrease in the number of lymphocytes (%; *p* < 0.05) and a highly significant decrease in all of the treatment groups (*p* < 0.01). Neutrophils and platelet distribution width (PDW; %) significantly decreased in the 40 mg/kg group (*p* < 0.05).

The serum biochemical parameters of rats in the four groups after 28 days of continuous intragastric administration of aurantio-obtusin are shown in Table 3. The serum levels of alanine transaminase (ALT), aspartate transaminase (AST), and alkaline phosphatase (ALP) in the high- and medium-dose groups were significantly higher than in the blank control group (*p* < 0.001). The AST and ALP in the low-dose group increased (*p* < 0.001, *p* < 0.01), but no significant difference in ALT was observed.

Compared with the control group, the total protein (TP) and albumin (ALB) levels in each group significantly decreased (*p* < 0.01, *p* < 0.001). The total cholesterol (TCHO) also decreased to varying degrees (*p* < 0.001).

### 2.3. Histopathological Examination of Liver

After 28 days of oral treatment with aurantio-obtusin, we dissected the rats and stained the liver sections with hematoxylin and eosin (H&E). We observed the results of histopathological examination under a microscope.

**Figure 2:**
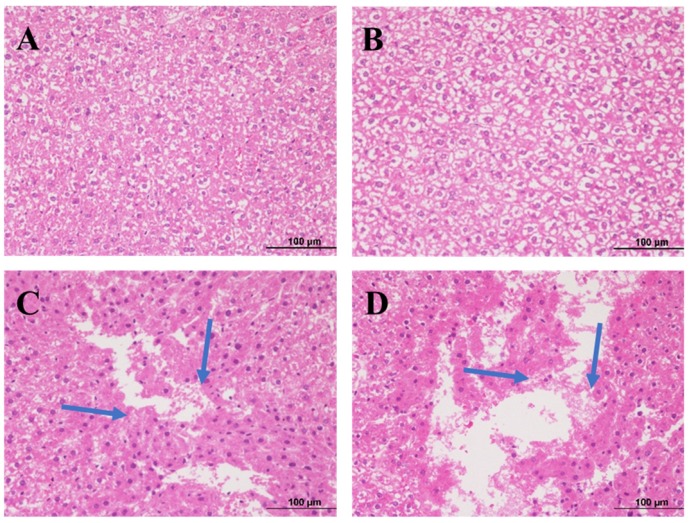
Histopathological observations of rat liver exposed to different doses of aurantio-obtusin and stained with hematoxylin and eosin (H&E): (**A**) vehicle control group; (**B**) low group; (**C**) medium group; and (**D**) high group. Each slide was examined at a magnification of 200 times. The blue arrow represents cell necrosis.

The morphology of hepatocytes in the low-dose group was normal, with mild degeneration and slight inflammatory infiltration (Figure 2B). We observed hepatocyte and vacuolar degeneration, inflammatory cell infiltration, and focal and flake necrosis in the medium-dose group (Figure 2C). We observed cytoplasmic loosening and vacuolar degeneration in hepatocytes, and the pathological changes were diffuse. We observed focal, patchy, and large patchy necrosis in the high-dose group (Figure 2D). In the blank group, the liver structure was clear, the hepatocyte morphology was normal, the hepatocytes were well arranged, and the structure of hepatic portal area was normal (Figure 2A).

### 2.4. Analysis of Serum Metabolites in Sprague–Dawley Rats with Aurantio-Obtusin-Induced Hepatotoxicity

#### 2.4.1. Methodology Validation

**Figure 3:**
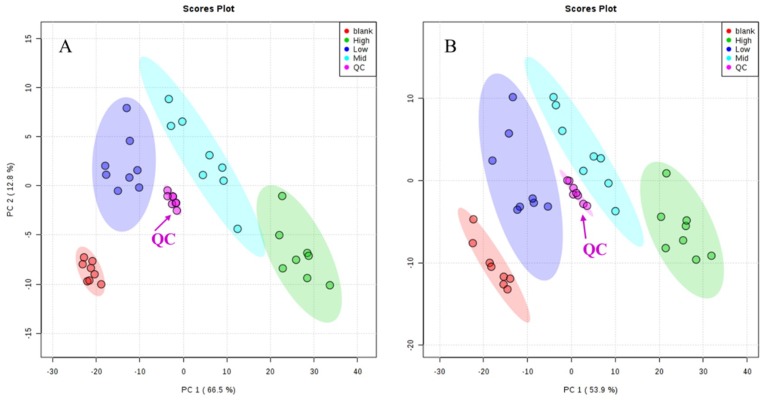
Principal component analysis (PCA) score plots of quality control (QC) samples in ES^+^ (**A**) and ES^−^ (**B**).

As shown in Figure 3, quality control (QC) samples were clustered in positive and negative ion modes. In addition, five ion signals were randomly selected to analyze their retention time and peak area in positive and negative ion modes. The relative standard deviations (RSD%) of retention time were 0~0.22% and 0~0.16% in positive and negative ion modes, respectively. The RSD% of the peak area were 2.08~5.25% and 2.68~8.96%, respectively (Table 4). The results of methodological validation showed that the method had good repeatability, high precision, and could be used for batch metabolomics analysis.

#### 2.4.2. Metabolic Spectrum of Rat Serum

**Figure 4:**
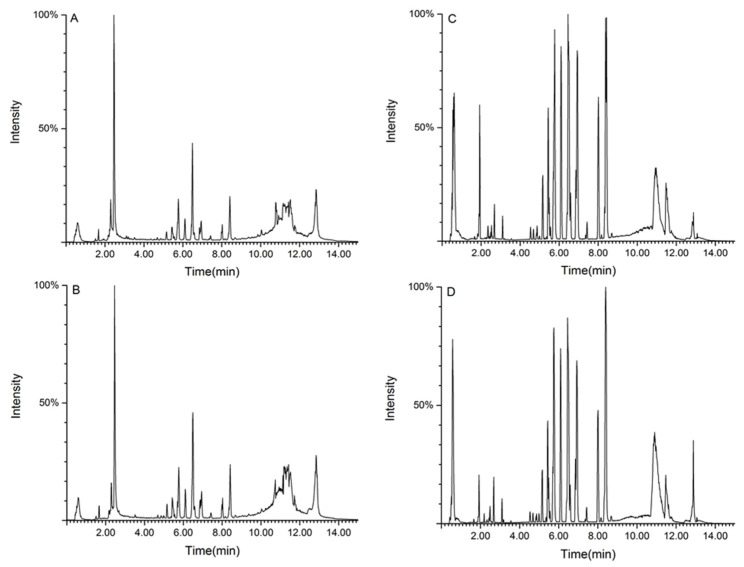
Total ion flow chromatography (TIC) of serum samples derived from rats in the control group (**A**,**C**) and the aurantio-obtusin (200 mg/kg) group (**B**,**D**). Positive (**A**,**B**) and negative (**C**,**D**) ion mode.

We used ultra-high performance liquid chromatography-quadrupole time-of-flight tandem mass spectrometry (UPLC-Q-TOF-MS/MS) to separate and collect serum samples. Figure 4 presents the results of total ion flow chromatography (TIC) in the positive and negative ion modes of rat serum in the control group and aurantio-obtusin-treated group.

#### 2.4.3. Metabolic Track Analysis of Rat Serum

After data preprocessing of all of the samples, we used TIC as a pattern recognition variable. Principal component analysis (PCA) and partial least squares discriminant (PLS-DA) were used to establish the metabolomic models of rats treated with normal and different doses of aurantio-obtusin. We analyzed the metabolic track using a score map and investigated the degree of deviation between the aurantio-obtusin group and the normal group to evaluate the overall hepatotoxicity.

**Figure 5:**
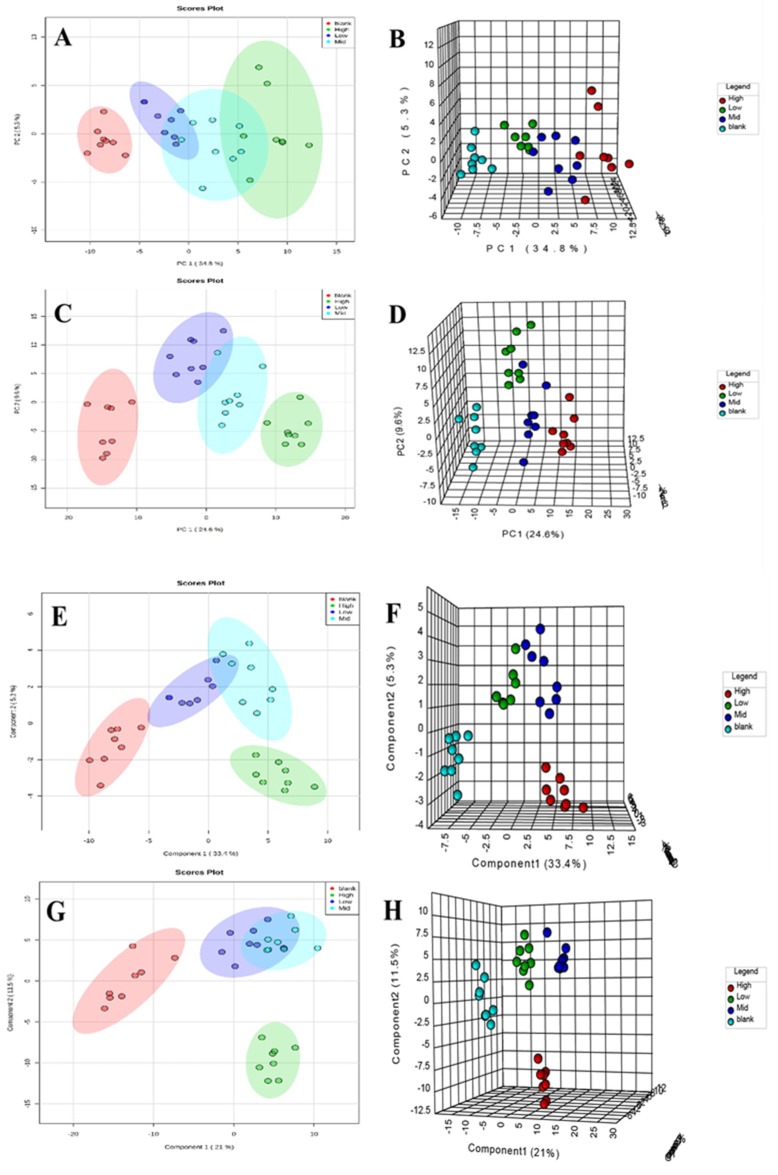
The PCA and partial least squares discriminant (PLS-DA) plots for separation of four different groups of rats after 28 days of exposure to aurantio-obtusin. Score map (**A**) and synchronized three-dimensional (3D) plot score (**B**) from PCA in positive ion mode. Score map (**C**) and synchronized 3D plot score (**D**) from PCA in negative ion mode. Score map (**E**) and synchronized 3D plot score (**F**) from PLS-DA in positive ion mode. Score map (**G**) and synchronized 3D plot score (**H**) from PLS-DA in negative ion mode.

The clustering and changing trend of each sample was visually displayed on the PCA score map (Figure 5A–D), and the blank and the aurantio-obtusin groups were clearly distinguished.

We established a metabolomic model of serum samples with different doses of aurantio-obtusin and the normal group through supervised PLS-DA (Figure 5E–H). As shown in Figure 5, rat serum metabolites changed significantly after continuous treatment with different doses of aurantio-obtusin. The changes in serum metabolites were distinct at different dosages. The samples containing different doses of aurantio-obtusin were separated from the blank samples, showing a very clear line of motion. The metabolic track was dose-dependent, and the metabolites varied significantly under different dosages after exposure to aurantio-obtusin, especially in the high-dose and control groups, suggesting toxicity after 28 days of high-dose treatment with aurantio-obtusin. The toxicity was increased with dosage. The metabolomic analysis of aurantio-obtusin is sensitive and rapid to facilitate early detection of hepatotoxicity in rats. The concentrations in the low- and medium-dose groups ranged between the high-dose and the blank groups. The high-dose group deviated most from the other three groups. The degree of deviation of the drug group was positively correlated with the dose of aurantio-obtusin. The degree of deviation of the high-dose group from the normal group was marked, which indicated that aurantio-obtusin altered the normal physiological state of rats in vivo. The significant variation in metabolites compared with the normal group suggested hepatotoxicity, which increased in severity with dosage. This result indicated that the dose-dependent hepatotoxicity correlated with the dosage of aurantio-obtusin.

#### 2.4.4. Identification of Biomarkers

**Figure 6:**
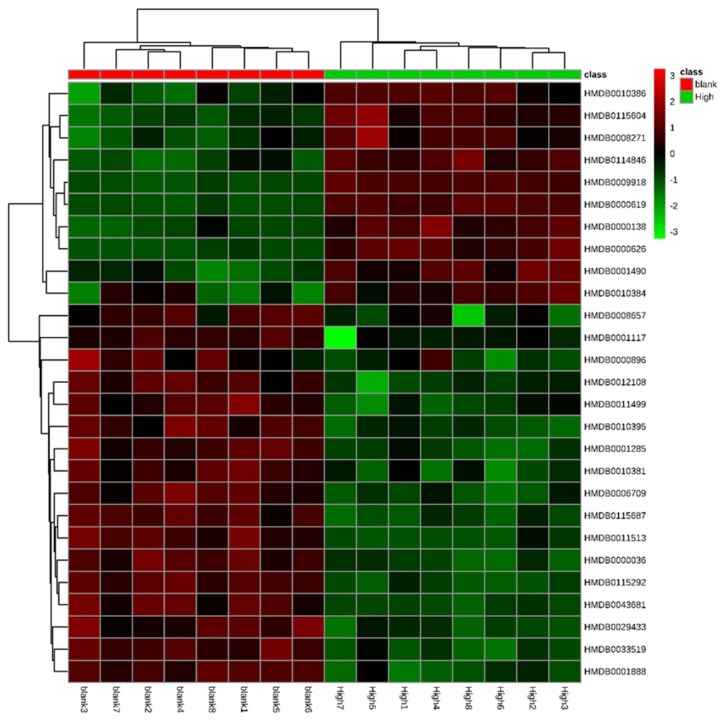
The serum metabolites of rats in the aurantio-obtusin group represent a dose-dependent heatmap. Each square represents the content of specific metabolites in each dose group. Green suggests a decrease in content, whereas red denotes an increase in content.

We measured the importance of variables in the classification according to their variable importance in projection (VIP) values and screened the variables accordingly. Differential metabolites with VIP >1.0 were screened, and the significant standard threshold was set at *p* < 0.05. The maximum variance among groups, the retention time, the mass-to-charge ratio, and mass spectrometry information were then determined. The public databases HMDB (http://www.hmdb.ca) and KEGG (http://www.genome.jp) were used for search and validation and were compared with relevant literature. We initially accepted molecules less than 5 ppm as potential biomarkers (Table 5). To visualize and effectively characterize the hepatotoxicity of aurantio-obtusin, we extracted 28 compounds, identified in Table 5, to construct a heatmap (Figure 6). We observed the fluctuations in the relative content of each metabolite in the rat serum sample with the dose changes during 28 days. The results showed that most lipids and amino acids were downregulated, and some bile acids were upregulated, suggesting that the metabolism of rats was disturbed by aurantio-obtusin treatment.

#### 2.4.5. Metabolic Pathway Analysis

**Figure 7:**
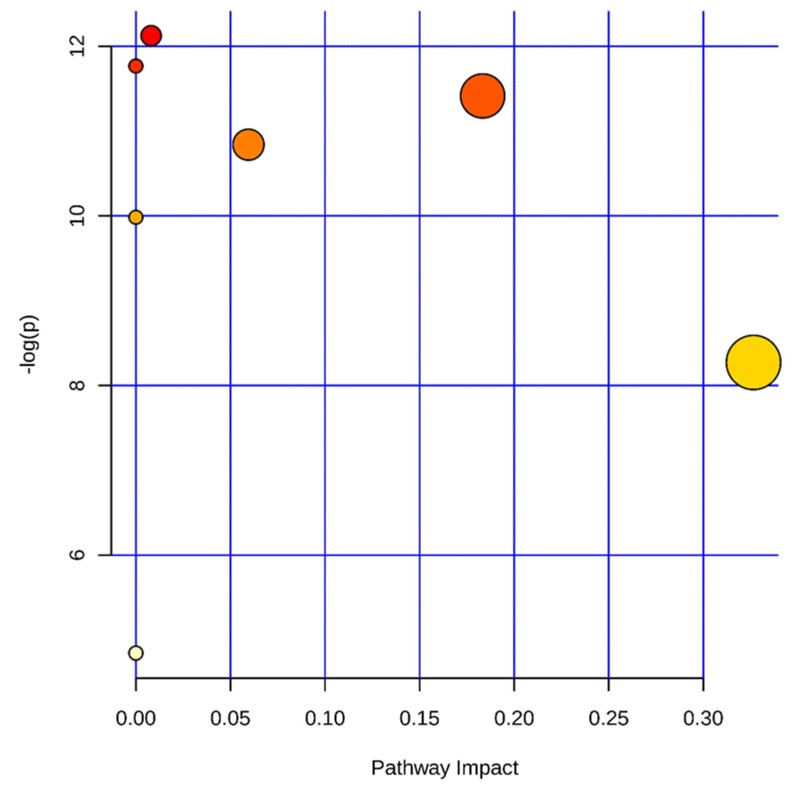
Summary of metabolic pathway analysis of different metabolites in rat serum induced by aurantio-obtusin. Log (*p*) value represents the color of the circle; impact represents the size of the circle, that is, the influence of the path.

**Figure 8:**
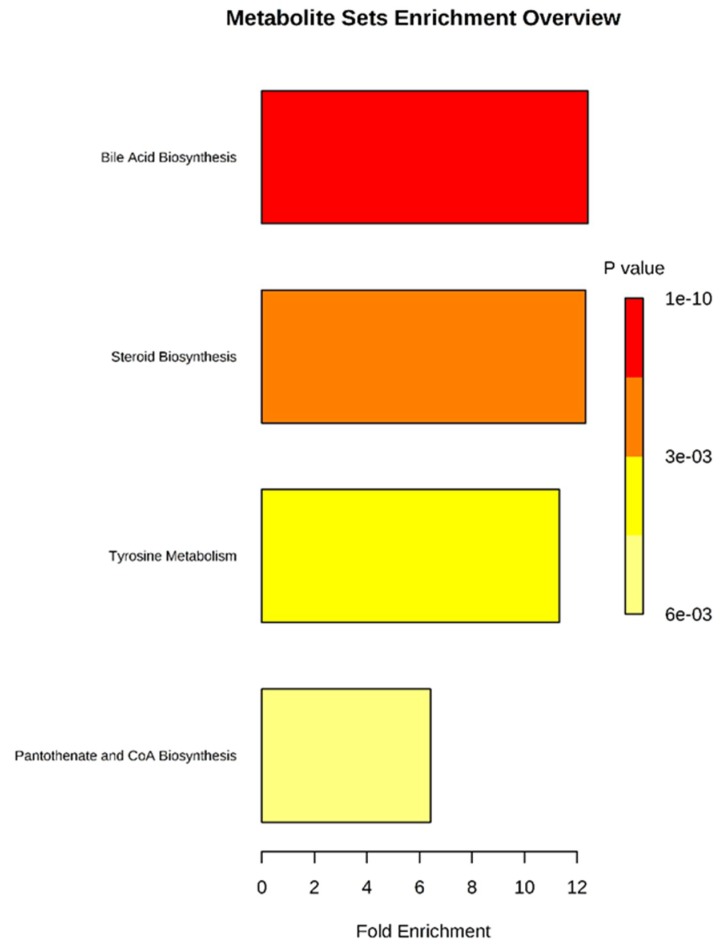
Metabolic pathway analysis of potential biomarkers related to liver injury in rats treated with aurantio-obtusin. The most relevant pathways are represented by large and dark nodes (pathway impact > 0.1).

**Figure 9:**
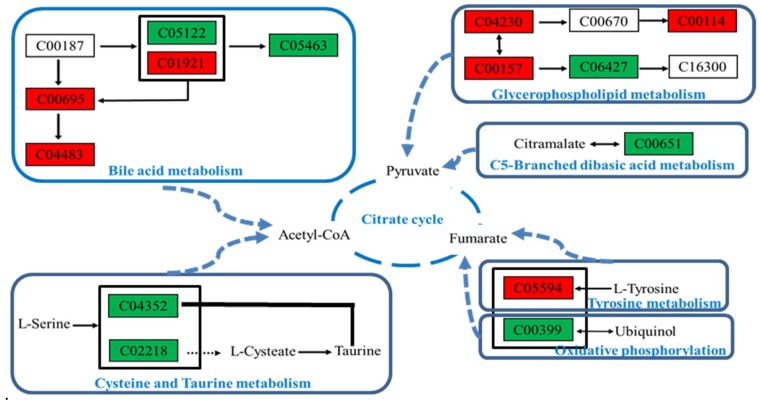
Toxicity mechanisms of the hepatotoxic metabolic network induced by aurantio-obtusin. Green denotes a decrease in content, whereas red indicates an increase in content.

The potential markers identified in Table 5 were entered into the MetaboAnalysis 4.0 (http://www.metaboanalyst.ca/) database. We selected metabolic pathways with a *p*-value of ≤0.05 or an impact ≥0.1 as potential targets, as shown in Figure 7. We identified four potential target pathways related to serum metabolites based on impact values and metabolite enrichment analysis (Figure 8). In particular, bile acid, steroids, tyrosine metabolism, and biosynthetic pathways of pantothenate and acetyl coenzyme A (CoA) were detected in serum and correlated with the identified metabolites, as shown in Figure 9. The metabolic pathways and the related toxicity mechanisms of the metabolic network of 28 potential biomarkers of hepatotoxicity induced by aurantio-obtusin are shown. The changes in metabolic levels of these potential biomarkers suggest that the hepatotoxicity may trigger disorders of bile acid, energy, and lipid metabolism. Further investigation into these pathways is essential to elucidate the underlying mechanism of hepatotoxicity.

## 3. Discussion

Aurantio-obtusin is an anthraquinone isolated from cassiae semen, for which it is listed as the quality control standard in the current Chinese pharmacopoeia. It is the main effective component of cassiae semen, with anti-inflammatory and hypolipidemic effects, as established in previous studies. However, in recent years, cases of liver injury caused by anthraquinones have been reported occasionally, which has led to public concern about the safety of anthraquinone-containing TCMs, especially those used as food and medicinal products. Because of the public’s awareness of non-toxicity, these products will be used as food for a long time in daily life. The clinical application of cassiae semen requires safety and toxicology studies of anthraquinone.

In this study, we analyzed the specific anthraquinone component in cassiae semen and evaluated its safety comprehensively and systematically. We tested the oral administration of aurantio-obtusin for 28 days at doses equivalent to the clinical dose (low-dose group), 10 times the clinical dose (medium-dose group), and 50 times the clinical dose (high-dose group). Combined with abnormal organ coefficient, abnormal hematology, serum biochemical parameters, and hepatocyte degeneration, the histopathological analysis of the three groups of rats showed a clustering of serum metabolite phenotypes after 28 days of continuous treatment with aurantio-obtusin based on metabolomics. We concluded that the symptoms of hepatotoxicity in rats were enhanced with the increased dosage of aurantio-obtusin. This result suggested that aurantio-obtusin should not be used for long-term therapy even at the usual clinical dosage.

Using metabolomic techniques, we analyzed the metabolism of endogenous small molecule substances in rats showing aurantio-obtusin-induced hepatotoxicity and elucidated the mechanism of intrinsic toxicity for metabolic disorders in vivo. We found that 28 endogenous metabolites (Table 5) were significantly different from those of normal rats after 28 days of oral treatment, suggesting disorders associated with the metabolism of bile acids, steroids, amino acids, and energy related to pantothenic acid and CoA synthesis.

Bile acid is synthesized from cholesterol via a series of endogenous substances in the liver. Normally, the metabolism of bile acid is in equilibrium. However, because of the inhibition of bile acid metabolism and excretion, cholestasis can cause liver and mitochondrial damage and can lead to apoptosis and hepatocyte failure. Therefore, changes in the bile acid metabolic network are of great significance in the study of liver injury mechanisms and the toxicity of TCM [23,24,25,26,27]. Experimental studies have shown that bile acids can be used as biomarkers to characterize hepatotoxicity. According to the results of PCA, PLS-DA, and related analyses, the hepatotoxicity of aurantio-obtusin may be related to cholestasis in rats and may also be positively correlated with the concentration of bile acids in serum, especially cholic acid, deoxycholic acid, and glycocholic acid. Cholic acid (CA) is the primary bile acid secreted in the liver. Deoxycholic acid (DCA) is a secondary bile acid produced by the liver. It is usually combined with glycine or taurine. It promotes fat absorption and cholesterol excretion, which also explains the lipid-lowering mechanism of aurantio-obtusin. The increased serum content in rats in the aurantio-obtusin group may damage the liver or hepatocytes, leading to a ruptured hepatocyte membrane. Glycocholic acid (GCA) is a combination of acyl glycine and bile acid glycine. In hepatocytes, primary and secondary bile acids bind to amino acids at the C-24 carboxylic acid on the side chain, so almost all of the bile acids in the bile duct exist in the glycine-bound form. The decrease of taurodeoxycholic and taurocholic acids (TDCA and TCA) may be attributed to liver injury induced by aurantio-obtusin, which disrupts the transformation of primary into secondary metabolic bile acid. Therefore, bile acids, such as CA, DCA, GCA, TCA, and TDCA, can be used as the primary biomarkers of hepatotoxicity induced by aurantio-obtusin in rats.

Lysophosphatidylcholine (LPC) is a phospholipid, which is metabolized primarily in the liver and is altered significantly in liver diseases and hepatotoxicity [28]. The steady state of several LPCs was disrupted in the aurantio-obtusin group, although with varying trends. After 28 days of treatment, the levels of serum LPC 18:2 and LPC 18:0 significantly increased, whereas the levels of LPC 17:0 and LPC 15:0 significantly decreased, suggesting that LPCs may be biomarkers of hepatotoxicity. Therefore, changes in LPCs in clinical practice should raise suspicion of liver injury. The type of LPCs that can be used as markers of hepatotoxicity induced by aurantio-obtusin require experimental confirmation.

Phosphatidylcholine (PC) is an important bioactive substance for hepatocytes to synthesize lipoproteins [29]. Lack of PC in the human body can lead to elevated ALT levels in the blood and liver function disorder.

Phosphatidylserine (PS) is a glycerol phospholipid and an active substance in the cell membrane [30]. Normally, it exists in the intracellular membrane. When aurantio-obtusin induces hepatocyte damage, it is translocated from the intracellular membrane to the outer membrane and eventually is released, which increases the blood content. Phosphatidylinositols (PI) are important lipids, both as a key membrane constituent and as a participant in essential metabolic processes. The reason for the increase of PI content may be the same as that of PS. Phosphatidic acid (PA) and Lysophosphatidylethanolamine (LysoPE) are a second messenger that regulates cell growth by regulating protein kinase, protein phosphatase, small G protein, and microtubule-related proteins [31,32]. LPC is a type of phospholipid with strong surface activity that ruptures erythrocytes and other cell membranes and induces hemolysis or cell necrosis. These changes in phospholipid content are not consistent, which indicates the diversity of molecular mechanisms associated with aurantio-obtusin-induced liver injury, which require further validation.

Ubiquinone-2 is a chemical compound known as polyprenylbenzoquinone. Ubiquinones are associated with cellular respiration. They are lipid-soluble, and therefore mobile in cell membranes, and play a unique role in the electron transfer chain.

Electrons from nicotinamide adenine dinucleotide and succinic acid enter oxygen via the electron transfer chain (ETC), which is then reduced to water. The electron transfer through the ETC results in H^+^ transport through the membrane and the proton gradient in the membrane, resulting in ATP synthesis [33,34]. It is a key substance in mitochondrial energy synthesis. The decrease of its content suggests that aurantio-obtusin may interfere with energy metabolism.

Vanylglycol is an O-methylated metabolite of norepinephrine [35]. Elevated levels of Vanylglycol may interfere with tyrosine metabolism, which in turn affects the tricarboxylic acid cycle through fumarate. It affects the citric acid cycle by disrupting the conversion of L-2-amino-4-methylene glutaric acid to citrate via pyruvate [36].

Geranyl diphosphate (Geranyl-PP) affects steroid biosynthesis to trigger disorders of bile acid metabolism [37], which in turn leads to liver damage.

Taurolithocholic acid 3-glucuronide is a natural human metabolite of taurocholic acid produced by uridine 5′-diphospho-glucuronosyltransferase (UDP-glucosyltransferase (UGT)) in the liver. Glucuronidation is used to eliminate toxins, drugs, or other substances that cannot be used as energy [38,39]. The decrease in taurolithocholic acid 3-glucuronide content indicates a decrease in the activity of UDP-glucosyltransferase, resulting in the accumulation of aurantio-obtusin, which affects liver function.

4′-Phosphopantothenoylcysteine (PPC) is an intermediate in the biosynthetic pathway that converts pantothenate (vitamin B5) into CoA [40]. CoA is the principal acyl carrier required in several synthetic and degradative reactions in intermediary metabolism and is an essential cofactor in all living systems. The decrease of PPC content interferes with CoA and affects the citric acid cycle.

Decreasing triglyceride (TG) levels may explain the role of aurantio-obtusin in lowering blood lipid levels [41], which is consistent with the reported pharmacological effects.

Enrichment and analysis of these endogenous markers indicate that the hepatotoxicity of aurantio-obtusin is mainly caused by the following pathways.

First, it interferes with bile and fatty acid metabolism and affects the tricarboxylic acid cycle via CoA. Second, it disrupts the biosynthetic pathway of steroids and affects the tricarboxylic acid cycle mediated via pyruvate. Third, it interferes with tyrosine metabolism and oxidative phosphorylation and affects the tricarboxylic acid cycle via fumarate. Finally, it affects the metabolism of cysteine and methionine and affects the tricarboxylic acid cycle mediated via CoA (Figure 9). 

## 4. Materials and Methods 

### 4.1. Chemicals, Reagents, and Materials

**Figure 10:**
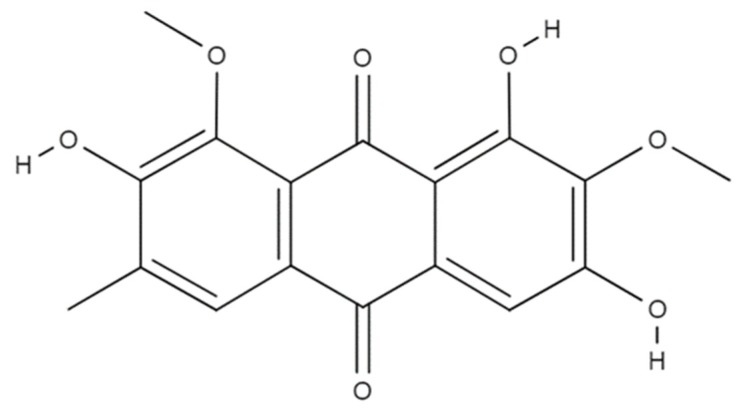
The molecular structure of aurantio-obtusin.

Aurantio-obtusin (CAS No. 67979-25-3, purity >97%), an anthraquinone (chemical structure shown in Figure 10), was obtained from Beijing Saibaicao Technology Co., Ltd., Beijing, China (Lot No. SH180806).

Methanol and acetonitrile were high performance liquid chromotography (HPLC) grade and purchased from Fisher Co. (Pittsburg, PA, USA). Formic acid (HPLC grade) was purchased from Merck (Darmstadt, Germany). Water was obtained from Watson’s Water (Watson, Hong Kong, China). Other chemicals were analytical grade.

Assay kits for the detection of hematological and biochemical indicators were purchased from Jiancheng Biological Technology, Co., Ltd. (Nanjing, China).

### 4.2. Animal Study Design

Male Sprague–Dawley rats (*n* = 32, 160 ± 20 g) were obtained from Charles River China Inc. (Vital River Laboratories, Beijing, China). They were randomly divided into four groups: (1) Control group (containing equal amounts of sodium carboxymethyl cellulose); (2) low-dose group (4 mg/kg); (3) medium-dose group (40 mg/kg); and (4) high-dose group (200 mg/kg). All of the animal experiments were performed in accordance with the guidelines of the European community, and approval (IACUC-DWZX-2018-010, October 2018) was granted by the Animal Care and Use Committee, Academy of Military Medical Sciences, Beijing, China.

The low, medium, and high doses of aurantio-obtusin corresponded to 1-, 10-, and 50-fold clinical doses, respectively. Each group of rats had intragastric administration once daily in the morning for 28 days. At the end of the experiment, rats were executed and the liver was extracted for analysis.

### 4.3. Sample Collection and Preparation

Blood was drawn from abdominal aorta after 28 days of continuous administration of aurantio-obtusin. Some serum samples were collected by centrifugalization and biochemical parameters were determined. The supernatant was centrifugalized for 10 min at 3000 rpm at 4 °C. The serum was frozen and stored until analysis (−80 °C). Before analysis, the serum samples were thawed at room temperature. After thawing, 200 μL serum were precisely measured. Protein was precipitated by adding three times the methanol solution. The supernatant was centrifugalized for 5 min (13,000 rpm) and the supernatant was transferred to an EP tube. The supernatant was dried by nitrogen at 4 °C. The residue was dissolved with 200 μL methanol for 60 s. The supernatant was centrifugalized for 15 min (13,000 rpm) and 100 μL of the supernatant was removed for UPLC-MS analysis. Another part of the blood samples was transferred to EP tubes pre-coated with ethylenediaminetetraacetic acid dipotassium (EDTA-2K) for hematological measurement. After blood collection, rat livers were collected and fixed in 10% neutral formalin. Fixed rat liver specimens were embedded in paraffin, sectioned at 5 mm, and stained with hematoxylin and eosin for histopathological examination.

### 4.4. Sample Analysis

#### 4.4.1. Hematology, Serum Biochemistry, and Histopathological Analysis

The whole blood was anticoagulated with ethylenediaminetetraacetic acid dipotassium (EDTA-2K), and the hematological parameters were measured using an automatic blood analyzer (SIEMENS ADVIA2120, Munich, Germany). Relevant hematological parameters were WBCs, red blood cells (RBCs), HGB, HCT, the blood platelet number (PLT), and the mean corpuscular volume (MCV).

The serum biochemical parameters were measured using an automatic biochemical analyzer (Hitachi7020, Tokyo, Japan). Relevant biochemical parameters were as follows: ALT, AST, ALP, TP, ALB, and TCHO.

For histopathological examination, liver tissues were fixed and preserved in 10% neutral formalin and embedded in paraffin after dehydration. The slices (5 mm in thickness) were observed after staining with H&E.

#### 4.4.2. Preparation of Quality Control Samples

A total of 100 μL serum was taken from each sample, and all of the serum samples were blended to obtain a mixed serum. A total of 200 μL serum was then taken from the mixed serum and was treated according to the method of 4.3 to obtain the quality control (QC) sample. QC samples were sampled five times before analysis. The QC sample was then injected once every 8 experimental samples. A total of 8 QC samples were injected throughout the experimental cycle.

#### 4.4.3. Metabolomics Analyses

Chromatographic conditions: A water Acquity UPLC^®^HSS T3 chromatographic column (1.8 μm 2.1 × 100 mm) was used with a column temperature of 45 °C. The binary solvent system included a mobile phase A containing 0.1% formic acid aqueous solution and a mobile phase B containing 0.1% formic acid acetonitrile solution. The gradient elution conditions were as follows: 0~1 min, 98% A; 1~2 min, 98~90% A; 2~11 min, 90~50% A; 11~13 min, 50~0% A; 13~14 min, 0~98% A; 14~15 min, 98% A. The flow rate was 0.5 mL·min^−1^ and the injection volume was 5 μL.

Mass spectrometric conditions were based on electrospray ionization (ESI), with an ion source temperature of 100 °C, a desolvent temperature of 450 °C, with a desolvent gas (N_2_) flow rate of 900 L·h^−1^. In the negative ionization mode, the capillary ionization voltage was 2.9 kV. In the positive ionization mode, the capillary ionization voltage was 3.0 kV; and the sampling cone hole voltage is 40 V. The mass scanning range was *m*/*z* 50–1000, and the scan time was 0.1 s. Under MS/MS mode, the collision energy varied between 10 and 30 eV. The TOF ion-flying mode was V mode, and leucopentin was used as an external standard for accurate mass determination of target ions.

### 4.5. Statistical Analysis

The MS raw data were converted into mzXML format using MS Convert software, and XCMS (www.bioconductor.org/) was used to extract the peak data, peak denoise, peak matching, peak alignment, and export. The results were saved for non-target metabolomics.

Before multivariate statistical analysis of variables, the exogenous components were removed, and the logarithmic (LOG) transformation and pareto-scaling were used to eliminate the differences in metabolic concentration by orders of magnitude. Automatic modeling and analysis were carried out. After data preprocessing, Excel tables, including sample identity (ID), molecular mass (MS), peak area, and standardized ion strength, were saved and derived to form a three-dimensional data matrix.

In each sample, the peak areas of metabolites were normalized using a sum method via the MetaboAnalyst software program (www.metaboanalyst.ca). A data set of all of the samples, including the retention time and normalized peak area of metabolites, was imported into MetaboAnalyst 4.0 software (version 4.0, McGill University, Montreal, Canada) for data pre-processing. Unsupervised PCA was used to evoke a general response to the clustering trend of metabolic profiles among groups. The supervised PLS-DA or orthogonal partial least squares discriminant (OPLS-DA) multidimensional statistical analysis was used to reduce data dimensionality, for sample classification and discrimination, and obtain the greatest difference between groups. A score plot of the OPLS-DA model was used to visualize the metabolic difference between the groups treated with drugs and the control. The potential biomarkers were preliminarily screened with a loading plot and variable importance in project. Data were analyzed by one-way analysis of variance (one-way ANOVA) to test the significance of differences between the control and aurantio-obtusin-treated groups. Significant differences were considered significant when test *p* values were less than 0.05. Multiple comparisons between the groups were performed using the Scheffe method. Finally, the variable with a significant difference was identified as the biomarker of potential hepatotoxicity induced by aurantio-obtusin. The significant metabolic biomarkers were screened using the exact molecular ion measured by MS/MS. The potential biomarkers were tentatively identified by matching the accurate mass charge ratio and MS/MS fragmentation patterns to the online database (5 ppm as the accepted mass error), such as the HMDB database (http://www.hmdb.ca). Finally, the metabolic pathways of the biomarkers of liver poisoning were analyzed using MetaboAnalysis 4.0 (http://www.metaboanalyst.ca), based on high-throughput metabolomics data. 

## 5. Conclusions

This study examined the hepatotoxic effects of aurantio-obtusin in rats after 28 days of oral treatment at different doses.

We have preliminarily confirmed that the intake of aurantio-obtusin at medium and high doses (40 mg/kg and 200 mg/kg, respectively) may lead to liver injury, indicated by the increased levels of ALT, AST, and ALP and by decreased TP, ALB, and TCHO activity. Different dosages of aurantio-obtusin affected hematological (HGB, HCT, WBC, NEUT, LYMPH, and PDW) parameters. The increased liver coefficient and the large number of pathological changes were related to long-term exposure to high doses of aurantio-obtusin. 

Our metabolomic analysis has successfully identified 28 potential endogenous metabolites, including those generated by the metabolism of bile acids, steroids, and amino acids, and energy metabolism associated with pantothenic acid and CoA synthesis, which may be related to liver injury induced by aurantio-obtusin.

Future research will focus on compounds related to the metabolism of bile acids, lipids, energy, and amino acids. The changes in endogenous bile acid content related to bile acid metabolism may contribute to hepatotoxicity induced by aurantio-obtusin treatment.

Thus, measuring the changes in serum could serve as the surrogates for the evaluation of hepatotoxicity. In addition, the potential toxicity mechanism can be elucidated through monitoring its metabolites profile. It provides a method for further explaining the toxicity mechanism of aurantio-obtusin and other anthraquinones. However, the main limitation of this work is the lack of quantitative and dynamic analysis of specific metabolites in oral aurantio-obtusin rats, which needs to be further validated by targeted metabolomics.

## Figures and Tables

**Table 1:** Body weight and organ coefficient of liver in each group after 28 days of continuous treatment with aurantio-obtusin.

Group	Body Weight/g	Liver	
		Weight/g	W/W*100(%)
0 mg/kg	339 ± 13.41	11.56 ± 1.36	3.41 ± 0.25
4 mg/kg	344 ± 11.54	12.18 ± 0.40	3.54 ± 0.34
40 mg/kg	334 ± 9.77	12.19 ± 0.51	3.65 ± 0.14 *
200 mg/kg	335 ± 8.32	13.71 ± 0.85 **	4.09 ± 0.26 ***

Compared with the 0 mg/kg group, * *p* < 0.05, ** *p* < 0.01, *** *p* < 0.001 (x ± s, *n* = 8).

**Table 2:** Hematological results of rats in each group after 28 days of continuous exposure to aurantio-obtusin. White blood cell (WBC); hemoglobin (HGB); hematocrit (HCT); platelet distribution width (PDW); blood platelet number (PLT); mean corpuscular volume (MCV); red blood cell (RBC); mean platelet volume (MPV); plateletcrit (PCT); mean corpuscular haemoglobin (MCH); mean corpuscular haemoglobin concentration (MCHC); neutrophilic granulocyte (NEUT); lymphocytes (LYMPH); monocyte (MONO); eosinophilic granulocyte (EO); basophilic granulocyte (BASO); reticulocytes (RET).

	Control	Aurantio-obtusin Concentration		
	—	4 mg/kg	40 mg/kg	200 mg/kg
WBC (10^9^/L)	5.44 ± 1.48	3.77 ± 0.90 *	3.08 ± 0.72 **	4.66 ± 1.75
RBC (10^12^/L)	8.47 ± 0.38	8.52 ± 0.38	8.10 ± 0.36	8.11 ± 0.37
HGB (g/dL)	167.88 ± 6.85	166.38 ± 8.43	159.86 ± 5.17 *	157.67 ± 5.56 **
HCT (%)	47.93 ± 1.70	47.24 ± 2.00	45.67 ± 1.51 *	45.32 ± 1.58 **
PLT (10^9^/L)	950.38 ± 97.89	953.50 ± 150.34	940.14 ± 90.91	981.00 ± 170.57
MPV (fL)	7.15 ± 0.24	7.13 ± 0.22	7.14 ± 0.18	7.28 ± 0.09
PCT (%)	0.68 ± 0.08	0.68 ± 0.11	0.67 ± 0.06	0.72 ± 0.12
MCV (fL)	56.63 ± 1.33	55.48 ± 1.47	56.40 ± 0.98	55.93 ± 1.77
MCH (pg)	19.84 ± 0.38	19.56 ± 0.54	19.76 ± 0.36	19.45 ± 0.53
MCHC (g/dL)	350.13 ± 4.65	352.38 ± 4.15	350.00 ± 2.39	347.67 ± 4.50
NEUT (%)	7.48 ± 2.83	15.49 ± 8.68 *	7.33 ± 2.44	6.09 ± 2.29
LYMPH (%)	90.14 ± 2.73	81.38 ± 8.75 *	90.13 ± 3.02	91.67 ± 2.63
MONO (%)	1.75 ± 0.83	2.25 ± 0.54	1.87 ± 0.60	1.60 ± 0.89
EO (%)	0.63 ± 0.27	0.86 ± 0.37	0.67 ± 0.38	0.65 ± 0.31
BASO (%)	0.01 ± 0.03	0.03 ± 0.07	0.00 ± 0.00	0.00 ± 0.00
NEUT#	0.39 ± 0.14	0.59 ± 0.30	0.23 ± 0.10 *	1.21 ± 1.44
LYMPH#	4.92 ± 1.40	3.06 ± 0.78 **	2.77 ± 0.62 **	3.33 ± 0.48 **
MONO#	0.09 ± 0.04	0.09 ± 0.03	0.06 ± 0.03	0.09 ± 0.08
EO#	0.04 ± 0.02	0.03 ± 0.01	0.02 ± 0.02	0.03 ± 0.02
BASO#	0.00 ± 0.00	0.00 ± 0.00	0.00 ± 0.00	0.00 ± 0.00
PDW (%)	8.00 ± 0.30	7.91 ± 0.29	7.73 ± 0.17 *	8.02 ± 0.27
RET#	0.10 ± 0.02	0.09 ± 0.03	0.09 ± 0.02	0.10 ± 0.02
RET (%)	1.22 ± 0.26	1.07 ± 0.34	1.07 ± 0.22	1.23 ± 0.24

Compared with the control group, * *p* < 0.05, ** *p* < 0.01, *** *p* < 0.001 (x ± s, *n* = 8).

**Table 3:** Serum biochemical indices of rats in each group after 28 days of continuous treatment with aurantio-obtusin. Alanine transaminase (ALT); aspartate transaminase (AST); alkaline phosphatase (ALP); total protein (TP); albumin (ALB); total cholesterol (TCHO).

	Control		Aurantio-Obtusin	
	—	4 mg/kg	40 mg/kg	200 mg/kg
ALT (U/L)	30.17 ± 5.34	32.50 ± 3.86	62.33 ± 5.76 ***	85.67 ± 7.13 ***
AST (U/L)	89.20 ± 10.93	115.17 ± 10.40 ***	189.63 ± 22.11 ***	223.17 ± 16.34 ***
ALP (U/L)	233.17 ± 23.05	288.67 ± 41.52 **	353.33 ± 44.43 ***	560.83 ± 60.88 ***
TP (g/L)	44.22 ± 3.85	35.64 ± 2.91 ***	36.70 ± 4.23 ***	33.25 ± 4.45 ***
ALB (g/L)	22.40 ± 1.90	19.75 ± 1.16 **	19.35 ± 1.84 **	17.43 ± 2.20 ***
TCHO (mmol/L)	1.48 ± 0.15	1.17 ± 0.07 ***	1.11 ± 0.08 ***	1.00 ± 0.13 ***

Compared with the control group, * *p* < 0.05, ** *p* < 0.01, *** *p* < 0.001 (x ± s, *n* = 8).

**Table 4:** The relative standard deviations (RSD) of T_R_ and the peak area in positive and negative mode.

Ion Mode	T_R__*m*/*z*	RSD (%)	
		T_R_	Area
Positive	1.98_317.0694	0.22	2.08
	5.75_620.2559	0	3.82
	6.10_240.1049	0.21	5.23
	8.01_323.2295	0	2.54
	11.68_369.3548	0.04	5.25
Negative	3.33_221.1546	0.08	3.05
	5.70_630.3229	0.14	2.68
	6.48_481.3134	0.12	3.34
	7.43_628.2766	0.16	5.52
	11.37_624.4236	0	8.96

**Table 5:** Preliminary identification of potential biomarkers in rat serum in the high-dose group of aurantio-obtusin. Electrospray ionization (ESI).

Mode	RT/min	*m*/*z*	Formula	Biomarker	HMDB	KEGG	Trend	ppm
ESI+	5.75	240.1046	C_7_H_17_N_3_O_4_S	*N*-Ornithyl-l-taurine	033519	Unknown	↓	5
	5.44	520.3421	C_26_H_50_NO_7_P	LysoPC (18:2)	010386	Unknown	↑	0
	0.62	74.0684	C_3_H_7_NO	*N*,*N*-Dimethylformamide	001888	C03134	↓	1
	12.87	778.5466	C_44_H_76_NO_8_P	PC (22:5/14:1)	008657	C00157	↓	2
	12.94	319.198	C_19_H_26_O_4_	Ubiquinone-2	006709	C00399	↓	1
	6.1	185.0824	C_9_H_12_O_4_	Vanylglycol	001490	C05594	↑	5
	6.94	794.5065	C_43_H_72_NO_10_P	PS (15:0/22:6)	112339	Unknown	↑	4
	8.41	524.3724	C_26_H_54_NO_7_P	LysoPC (18:0)	010384	C04230	↑	2
	13.12	811.6233	C_47_H_87_O_8_P	PA (24:1/20:2)	115604	Unknown	↑	1
	12.79	812.6262	C_46_H_86_NO_8_P	PC (20:0/18:3)	008271	C00157	↑	4
	0.59	160.0674	C_6_H_9_NO_4_	l-2-Amino-4-methylenepentanedioic acid	029433	C00651	↓	3
ESI-	12.86	883.5334	C_47_H_81_O_13_P	PI (22:5/16:0)	009918	C00626	↑	5
	12.87	803.5625	C_47_H_81_O_8_P	PA (22:1/22:5)	115292	Unknown	↓	0
	0.56	313.066	C_10_H_20_O_7_P_2_	Geranyl-PP	001285	C05847	↓	4
	5.75	507.3172	C_25_H_49_O_8_P	PA (8:0/i-14:0)	115687	Unknown	↓	2
	8.01	508.3416	C_25_H_52_NO_7_P	LysoPC (17:0)	012108	C04230	↓	2
	12.85	723.5049	C_41_H_73_O_8_P	PA (16:0/22:4)	114846	Unknown	↑	4
	8.34	480.3101	C_23_H_48_NO_7_P	LysoPC (15:0)	010381	C04230	↓	1
	5.72	504.3108	C_25_H_48_NO_7_P	LysoPE (20:2/0:0)	011513	Unknown	↓	1
	6.42	658.3331	C_32_H_53_NO_11_S	Taurolithocholic acid 3-glucuronide	002429	Unknown	↓	5
	5.7	552.3101	C_29_H_48_NO_7_P	LysoPE (0:0/24:6)	011499	Unknown	↓	1
	2.61	401.0877	C_12_H_23_N_2_O_9_PS	4′-Phosphopantothenoylcysteine	001117	C04352	↓	3
	12.88	857.6731	C_56_H_90_O_6_	TG (15:0/18:4/20:5)	043681	Unknown	↓	4
	1.94	407.2812	C_24_H_40_O_5_	Cholic acid	000619	C00695	↑	2
	6.97	464.3017	C_26_H_43_NO_6_	Glycocholic acid	000138	C01921	↑	0
	5.08	498.2874	C_26_H_45_NO_6_S	Taurodeoxycholic acid	000896	C05463	↓	4
	3.19	391.2853	C_24_H_40_O_4_	Deoxycholic acid	000626	C04483	↑	0
	2.32	514.2822	C_26_H_45_NO_7_S	Taurocholic acid	000036	C05122	↓	4

Note: Trend: Compared with the normal group, the content of compounds in the aurantio-obtusin group increased (↑) and decreased (↓). The human metabolome database (HMDB) is the human metabolism group database, column to 000036, for example, on behalf of this compound in the HMDB number; Kyoto encyclopedia of genes and genomes (KEGG) is a database of gene function analysis, KEGG column to C05122, for example, C for compound, 05122 on behalf of this compound in the KEGG number. Phosphatidylcholine (PC).
